# Biodegradable Active Packaging Enriched with Essential Oils for Enhancing the Shelf Life of Strawberries

**DOI:** 10.3390/antiox12030755

**Published:** 2023-03-20

**Authors:** Magdaléna Rusková, Alena Opálková Šišková, Katarína Mosnáčková, Custódia Gago, Adriana Guerreiro, Mária Bučková, Andrea Puškárová, Domenico Pangallo, Maria Dulce Antunes

**Affiliations:** 1 Institute of Molecular Biology, Slovak Academy of Sciences, Dúbravská cesta 21, 845 51 Bratislava, Slovakia; 2 Polymer Institute of Slovak Academy of Sciences, Dúbravská cesta 9, 845 41 Bratislava, Slovakia; 3 Institute of Materials and Machine Mechanics, Slovak Academy of Sciences, Dúbravská cesta 9, 845 13 Bratislava, Slovakia; 4 Mediterranean Institute for Agriculture, Environment and Development & CHANGE—Global Change and Sustainability Institute, FCT, Universidade do Algarve, edf. 8, Campus de Gambelas, 8005-139 Faro, Portugal; 5 Centre for Electronics, Optoelectronics and Telecommunications, FCT, Universidade do Algarve, edf. 8, Campus de Gambelas, 8005-139 Faro, Portugal

**Keywords:** active packaging, PLA/PHB, essential oils, *Fragaria ananassa*, shelf life, antioxidant, antimicrobial

## Abstract

The strawberry (*Fragaria ananassa*) is a nutrient-rich fruit with high content of health-beneficial compounds. However, strawberries are susceptible to mechanical damage and microbiological contamination which can cause changes in fruit sensory properties. These changes consequently effect on ripening and shelf life of the strawberry. In recent years, essential oils (EOs) have been famous for their antimicrobial and antioxidant properties and are promising ecological alternatives to chemical antimicrobial substances. Nowadays, active packaging is one of several techniques developed for slowing down the metabolic processes of fresh fruits. Poly(lactic acid) (PLA) is one of the several polymers suitable for encapsulation EOs, whereas at the same time represent non-toxic, biodegradable, and compostable polymer derived from renewable resources. Suitable packaging prolongs the shelf life of fruit, keeps the products at the highest possible nutrition level, improves quality, and attracts customer attention. In the current study, we encapsulated EOs (lemongrass and oregano) into a PLA and poly(3-hydroxybutyrate) (PHB) packaging film and explored their antimicrobial and antioxidant properties. Moreover, biochemical and quality parameters for strawberry preservation and shelf-life extension were also assessed. Our tested active packaging film with EOs was proven to be useful for postharvest quality maintenance and shelf-life extension of strawberries, with PLA/PHB/ATBC + 5% lemongrass EO being slightly better than PLA/PHB/ATBC + 5% oregano EO.

## 1. Introduction

Food waste has become a central topic in environmental and economic aspects as well as an ethical topic since more than 820 million people globally suffer from hunger and malnutrition [1]. Considering an increasing population and shifting dietary habits, the demand for fruits has risen in recent decades [2]. The most suitable strategy for reducing food waste seems to be increasing and extending the shelf life and safety of food. The high perishability of fruit and vegetables affects the volume of active compounds in foods such as antioxidants, anthocyanin, and phenols, which affect color, texture, taste, and shelf-life, causing a loss of nutritional value and safety for consumers and leading to loss of freshness, rapid food spoilage, and change in the organoleptic properties of foods [3]. Additionally, microbial contamination causes food deterioration to a large extent [4].

Several techniques for slowing down the metabolic processes of fresh fruits have been developed to reach long-term storage, such as modified atmosphere packaging, active packaging, and edible coatings [2]. In this context, active food packaging represents a promising research area in the academic and industrial areas [5]. The other alarming problem in recent years presents the over-used plastic materials for food packaging. Currently, in society, there is increasing interest in fully biodegradable “green” materials, mainly due to the accumulation of plastic waste in landfills and its penetration and contamination in the form of microplastics into the whole ecosystem, especially in water [6]. Biobased materials are the new trends in food packaging to improve biodegradability. According to Mosnáčková et al. [7], the biodegradable polymers represent materials which easily undergo enzymatic hydrolysis, and they are prone to natural recycling by biological processes. The aliphatic polyesters are the most attractive polymeric material because of their good mechanical properties, processability, and the ability to undergo both the hydrolytic degradation and biodegradation by soil microorganisms in compost. Poly(lactic acid) (PLA) represents non-toxic, biodegradable, and compostable polymers derived from renewable resources. Blending PLA with other polymers is an inexpensive approach that can lead to the synergistic effect resulting in greatly improved mechanical properties [6,8].

Great attention has been dedicated to PLA due to its thermoplastic behavior, biocompatibility and physical properties, and the ability to improve these properties by the addition of active ingredients [3,9,10]. In our case, it was the inclusion of essential oils (EOs), especially oregano and lemongrass. EOs are natural antimicrobial agents extracted from aromatic plants by distillation. They are a very complex mixture of compounds belonging to different chemical classes (hydrocarbons, alcohols, esters, ethers, aldehydes, ketones, phenols), possessing numerous biological activities [11,12,13]. EOs are known by their antibacterial, antifungal, antioxidant, anti-inflammatory, and insecticidal properties [14,15]. Due to their multi-component natures, the antimicrobial mechanism of EOs is multitarget, and up to now, there is no evidence of the occurrence of EOS resistance [12,16].

Their large-scale industrial application has up to now been limited by the problems linked to the volatility, hydrophobia, and degradability of phytocomplexes [17]. However, electrospinning is one of the most effective and easy-used methods for encapsulation of EOs [18]. Electrospun fibers with encapsulated EOs provide the controlled release of the EOs from the polymer matrix to the food surface, prolonging its shelf-life, increasing safety, and preserving the quality [3].

One of the nutrient-rich and important small fruits with high content of vitamin C, vitamin E, vitamin B6, riboflavin, and other beneficial phytochemicals is strawberries (*Fragaria ananassa*). They are also biologically important for their antioxidant properties [19]. Strawberries are one of the fruits that are subject to rapid spoilage either by mechanical damage, microbial contamination, or softening in texture [2].

In this study, eco-friendly PLA/PHB packaging membranes with effective antimicrobial and antioxidant properties, conferred by the addition of EOs, have been developed. A PLA/PHB blend (85:15) was plasticized by acetyl tributyl citrate (ATBC) and filled with oregano or lemongrass EOs. These biodegradable membranes were applied to the packaging of strawberries in order to ensure decreased spoilage and prolong their shelf life. The new-developed packaging membranes were evaluated for their abilities to assure the quality and safety of packaged fruit and consequently the health and satisfaction of consumers.

## 2. Materials and Methods

Commercial poly(lactic acid) pellets PLA 4042 D were provided by Resinex Slovakia and manufactured by Nature Works^®^ with density 1.25 g/cm^3^. Poly (3-hydroxybutyrate) PHB Biomer^®^ powder was supplied from Biomer (Krailling, Germany) with density 1.20 g/cm^3^. The plasticizer acetyl tributyl citrate (ATBC) 98%, was purchased from Sigma ALDRICH. 1,1,1,3,3,3-hexa fluoro-2-propanol (HFIP, >99.0% purity) was purchased from TCI Tokyo Kasei (Tokyo, Japan), and dichloromethane p.a. (DCM, 99.8% purity) was purchasedfrom Lach-Ner (Bratislava, Slovakia). Both of them were used for preparing solutions for electrospinning. 

Oregano (*Origanum vulgare*) and lemongrass (*Cymbopogon flexuosus*) essential oils were purchased from dōTERRA Ltd., Huntingdon, UK. The chromatograms and the peak reports of both essential oils are described in the Supplementary Materials.

Fresh strawberries (cultivar Palmeritas) were collected from a local agro-industry in Algarve region in Portugal from April to May during commercial ripening. All strawberries were harvested on the same day and selected according to their shape, color, and absence of physical damage or fungal infection.

### 2.1. PLA/PHB/ATBC Blend Preparation

The PLA/PHB/ATBC 80:20:15 blend was prepared by melt mixing in a Plasti Corder Brabender (Duisburg, Germany) at 175 °C at 40 rpm (rounds per minute) for 10 min.

### 2.2. Preparation of Polymer Solutions for Electrospinning

PLA/PHB/ATBC solution was prepared in concentrations 10% (*w*/*v*) in the blend of solvents 1,1,1,3,3,3-hexa fluoro-2-propanol (HFIP) and dichloromethane (DCM). PLA/PHB/ATBC was weighted into the vials and then the solvent HFIP was added into the vial. The solution was stirred on magnetic stirrer IKA (IKA^®^-Werke GmbH & Co. KG, Staufen, Germany) with intensity of 750 mph. After dissolving in HFIP, the DCM was added to obtain the required concentrations. Then, the oregano EO or lemongrass EO (5% (*v*/*v*) with respect to the volume of PLA/PHB/ATBC solution) was added to the PLA/PHB/ATBC solution and stirred 10 min under magnetic stirring to obtain homogenous solution. 

A GC/MS analysis of EOs was provided by the producer, who guaranteed the chemical composition of each EO. The EOs were stored in amber glass vials and sampled using sterile pipet tips to minimize contamination and oxygen exposure.

### 2.3. Electrospinning

Electrospinning was the method used for preparation of nanofibrous mats. Electrospinning was carried out under ambient temperature 23 ± 1 °C, with the humidity H = 47 ± 1%. The device was set up in a horizontal spinning configuration with a flat-end needle with a 0.8 mm inner diameter. Distance between collector and end of the needle was 13 cm. The applied voltage was 18 kV, with positive polarity. High voltage was generated by a high-voltage power supply Spellman SL-150W (Spellman, Bochum, Germany). The solution was fed by single-syringe pump model NE-1000 (New Era Pump Systems, Inc., Farmingdale, NY, USA) during electrospinning process, and the feeding rate was 1 mL/h. The electrospun fibers were collected on the flat collector covered by an aluminum foil. 

The nanofibrous membranes were designated as pure PLA/PHB/ATBC, PLA/PHB/ATBC + 5% oregano EO and PLA/PHB/ATBC + 5% lemongrass EO. These membranes were stored in plastic bags to minimize oxygen exposure, contamination, and loss of EO concentration. 

### 2.4. Scanning Electron Microscopy (SEM)

The morphology of electrospun membranes was observed by scanning electron microscopy (SEM), JSM Jeol 6610 microscope (JEOL, Tokyo, Japan) at accelerated voltage 15 kV. The samples were sputtered with a thin layer of gold by sputter coater Balzers SCD 040 (Balzers Union Limited, Balzers, Liechtenstein) to protect the non-conductive materials. Gold acts as the channel for the removing of the charged electrons from the material. AzTec (Springfield, NJ, USA) software was used for collecting SEM images. The images were post-processed using ImageJ software (LOCI, University of Wisconsin, Madison, WI, USA, freely available). The 300-fiber segments were analyzed randomly in 3 independently prepared samples to obtain a mean diameter for each type of nonwoven material, including the distribution of the fiber diameters. 

### 2.5. Attenuated Total Reflectance—Fourier Tranform Infrared Spectrometry Analysis (ATR—FTIR)

For Fourier transform infrared spectra recording, the spectrophotometer Nicolet 8700 (Thermo Fisher Scientific, Madison, WI, USA) with DTGS TEC detector was used. The spectra were recorded in the absorbance mode in region 4000–600 cm^−1^ at a resolution of 4 cm^−1^.

### 2.6. Thermogravimetric Analysis (TG)

Thermal analyzer Linseis L75/L81/2000 (Linseis Messgeraete GmbH, Selb, Germany) was used for thermogravimetric analysis. The investigated samples (approx. 20 mg) loosely filled a cylindrical crucible with the height of 14.0 mm and diameter of 6 mm. The analyses were performed in the inert atmosphere. The nitrogen was used with the flow of 12 L/h. The temperature was increased from 30 °C up to 500 °C with the rate of heating and then cooling of 10 °C/min.

### 2.7. Application to Active Packaging of Strawberry

**Figure 1:**
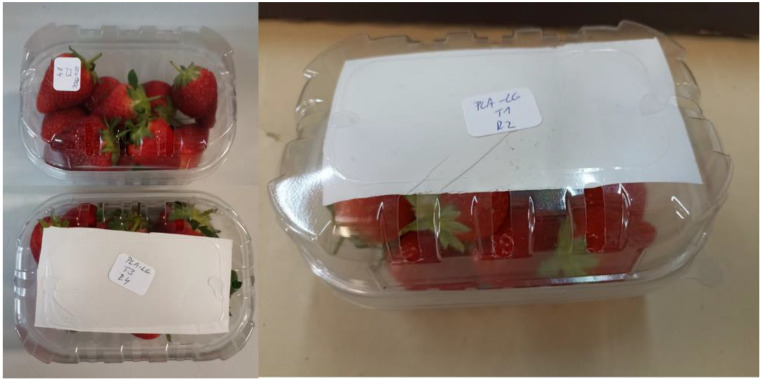
The application of biodegradable nanofibrous membranes (PLA/PHB/ATBC + 5% EO) in active packaging of strawberries.

In our experiment, representative fruits of the strawberry (cultivar Palmeritas) were selected to evaluate the application of biodegradable nanofibrous membranes (PLA/PHB/ATBC + 5% oregano EO and PLA/PHB/ATBC + 5% lemongrass EO) in active food packaging. Strawberries were selected and randomly allocated to the polyethylene terephthalate (PET) boxes with dimensions of (13  ×  10  ×  5 cm) and covered with their lids and kept in storage at 4  ±  0.5 °C and 85  ±  5% RH for 21 days. The experimental treatments included: (1) control: the fruit packed in PET container without film on its lid; (2) PLA/PHB/ATBC + 5% oregano EO: fruit packed in PET containers including pure PLA/PHB/ATBC + 5% oregano EO on the package lids (Figure 1); and (3) PLA/PHB/ATBC + 5% lemongrass EO: fruit packed in PET containers including PLA/PHB/ATBC + 5% lemongrass EO on the lids. All tested groups consisted of 4 replicates (4 PET containers with 10 fruits in each). Strawberries were tested for physicochemical properties on the initial day and then at 7-day intervals.

### 2.8. Quality Parameters

Quality parameters of strawberries were tested according to Gago et al. [20]. Color was recorded with a Chroma meter CR-300 series (CE Minolta, Osaka, Japan) and quantified by CIE (L*, a*, b*) color space. The lightness (L*) value ranges from black = 0 to white = 100, a* changes from green (negative values) to red (positive values), and b* from blue (negative values) to yellow (positive values). The a* and b* coordinates were converted to chroma (C*) (degree of departure from grey toward pure chromatic color) by the formula C* = (a*2 + b*2) 1/2 and hue angle (h°) (0° = reddish-purple, 90° = yellow, 180° = bluish-green, 270° = blue) by the formula h° = arctan (b*/a*).

Firmness was measured by puncture with a Chatillon TCD200 and a Digital Force Gauge DFIS50 (Jonh Chatillon&Sons, Inc., Somerset, FL, USA) fitted with a 7 mm diameter probe at a depth of 7 mm. Fruit firmness was measured in the middle of the strawberry fruit, and the results are expressed based on the average of 10 replicates (n = 10).

The soluble solids content (SSC) was measured in the fruit juice by a digital refractometer PR-1 ATAGO Co. LTD (Tokyo, Japan) in % (°Brix). 

Weight loss was expressed as a percentage of the initial weight.

### 2.9. Total Phenolic Content and Antioxidant Activity 

Extraction of total phenols and other active antioxidants was performed for each sample; 10 g of strawberry was squeezed with an UltraTurrax T 18 (IKA, Staufen, Germany) for 2 min, then centrifuged 5 min at 5000 rpm. The antioxidant activity of the extract was measured according to the modified method of Re et al. [21] and expressed as Trolox equivalent (µM Trolox.g^−1^ FW). Absorbance measurements were read at 515 nm after 20 min of incubation time at room temperature (A1). Absorbance of a blank sample containing the same amount of methanol and DPPH solution acted as the negative control (A0). The percentage inhibition [(A0 − A1/A0) × 100] was calculated and plotted against the Trolox standard curve [22]. The total phenolic content (mg GAE.g^−1^) was determined using the Folin–Ciocalteau reagent and gallic acid used as standard, as described by Slinkard and Singleton [23]. The sample (0.2 mL)) and 0.8 mL of sodium carbonate (75 g/L) were added to 1 mL of 10% (*w*/*v*) Folin—Ciocalteau reagent. After 30 min of reaction at room temperature, absorbance was measured at 765 nm [24].

### 2.10. Total Anthocyanin Content

Total anthocyanin content was calculated using the pH differential method according to Sun et al. [25] and Saldaña et al. [26]. The acidic buffer solution (pH 1) was prepared by mixing 0.186  g of KCl to 100  mL of distilled water, and the pH was adjusted to 1.0  ±  0.05 using HCl. The second solution (pH 4.5) was prepared by mixing 5.443  g of sodium acetate trihydrate with 100  mL of distilled water, and the pH was also adjusted with HCl. Liquid extracts of strawberry were mixed with buffer solution in a ratio of 1:1  *v*/*v* of extract:buffer. It was performed with a dilution of 1:50. Absorbance (A) of the diluted extracts was measured with a spectrophotometer at two wavelengths of 520 and 700  nm for each solution at pH 1 and pH 4.5. Anthocyanin content was calculated according to the following formula:

(1) $$Anthocyanincontent\left(\frac{mg}{L}\right)=\frac{A\times MW\times DF\times 1000}{\varepsilon \times 1}$$

where A =  (A520–A700  nm) pH 1.0—(A520–A700  nm) pH 4.5, molecular weight (MW) =  449.2  g/mol of Cy3GE (cyanidin-3-glucoside equivalent), dilution factor (DF) = 50, ε  =  26,900 molar extinction coefficient (L/mol cm of Cy3GE), 1 is the path length, and 10^3^ is the conversion of g to mg. Anthocyanin content was expressed as milligrams of Cy3GE per liter of the extract.

### 2.11. Microbiological Analysis

The microbiological quality of strawberry samples was determined by counting aerobic mesophilic bacteria, psychrotrophic bacteria [27], and yeasts and molds [28]. Ten grams of each sample was transferred to 90 mL of peptone water (Oxoid) and homogenized by a homogenizator classic/panoramic (IUL Instruments, Barcelona, Spain) and consequently diluted 10 times. To determine the counts of yeasts and molds, Chloramphenicol Glucose Agar (Biokar, Paris, France) was used, and to determine psychrotropic and aerobic mesophilic bacteria, Plate Count Agar (Biokar, Paris, France) was used. The incubation temperature of agar plates for yeasts and molds was 25 ± 1 °C for 48–72 h, 30 ± 1 °C for 24–72 h for aerobic mesophilic bacteria and 6.5 ± 1 °C for 5 to 10 days for psychrotrophic bacteria. Results were expressed as Log10 colony-forming units (CFU) per gram fresh weight [29].

### 2.12. Sensory Evaluation

Twenty people recruited from academic staff and students familiar with performing taste panels were used to evaluate the sensory parameters of strawberry (fruit appearance, pulp appearance, aroma, texture, sweetness, acidity, flavor in general) on a 7-point hedonic scale (1-bad; 7-excellent) after 18 days of storage.

### 2.13. Statistical Analysis

The experimental design was a complete randomized block design. Statistical analysis was performed using SPSS 24.0 software (IBM, Inc., Armonk, NY, USA). Factors as treatments and storage time were used for two-way analysis of variance (ANOVA). Duncan’s multiple-range tests (*p* < 0.05) for means comparison were performed.

## 3. Results and Discussion

Because of the novelty of the produced fibrous composites, the comprehensive characterization of the composite is needed. Therefore, the morphology of the fibrous webs by scanning electron microscopy, chemical composition by ATR-FTIR, and thermal analysis by thermal gravimetry were measured.

### 3.1. SEM

**Figure 2:**
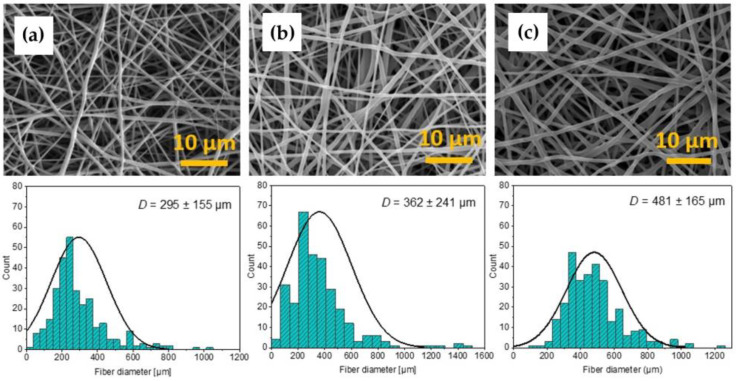
SEM images of the neat PLA/PHB/ATBC (**a**), PLA/PHB/ATBC + 5% oregano EO, (**b**) and PLA/PHB/ATBC + 5% lemongrass oil (**c**) fibers and matching histograms of fiber diameter distribution.

The plain PLA/PHB/ATBC, PLA/PHB/ATBC+5 oregano EO and PLA/PHB/ATBC + 5% lemongrass EO solutions were electrospun to obtain the fibrous membranes. The morphologies of the plain and EO-loaded PLA/PHB/ATBC fibrous membranes are shown in Figure 2. It can be observed that the plain PLA/PHB/ATBC membrane exhibits mainly spherical bead-free fibers in the area inspected by SEM. The addition of EO caused a higher representation of fibers with a flat cross-section, which could be entitled as “ribbon”. This may have several reasons. (i) It may be related to an expected increased viscosity with the addition of EOs as such structures were occasionally produced at a higher concentration, i.e., at a higher viscosity of electrospun polymer solutions. Authors observed it in studies of PA11 [30], PA6 [31], or PLA/PHB [32]. (ii) The immiscibility of the EOs and the polymer solution can also play a role. Immiscibility results in an emulsion, and fibers are produced from the emulsion [33]. (iii) The charge distribution can also cause ribbon formation during electrospinning when the thin skin is formed on the still-liquid polymer solution in the jet. An electrical charge distributed uniformly on the cylindrical jet tends to flow to the edges of the ribbon, producing a lateral force that favored the collapse [34]. The average diameters of the fibers increased with the addition of EOs. The matching histogram of the fibers diameter distribution for the investigated fibrous membranes are shown in Figure 2 as well. Average fibers diameters with the addition of 5% of oregano EO rose more than 20%, and with 5% of lemongrass EO they rose by more than 60%. The mentioned collapse of the circle cross-section of fibers can explain higher fiber diameters. It is supposed that differences in the average fiber diameter of PLA/PHB/ATBC + 5% oregano EO and PLA/PHB/ATBC + 5% lemongrass EO lies in the composition of EOs.

### 3.2. FTIR

**Figure 3:**
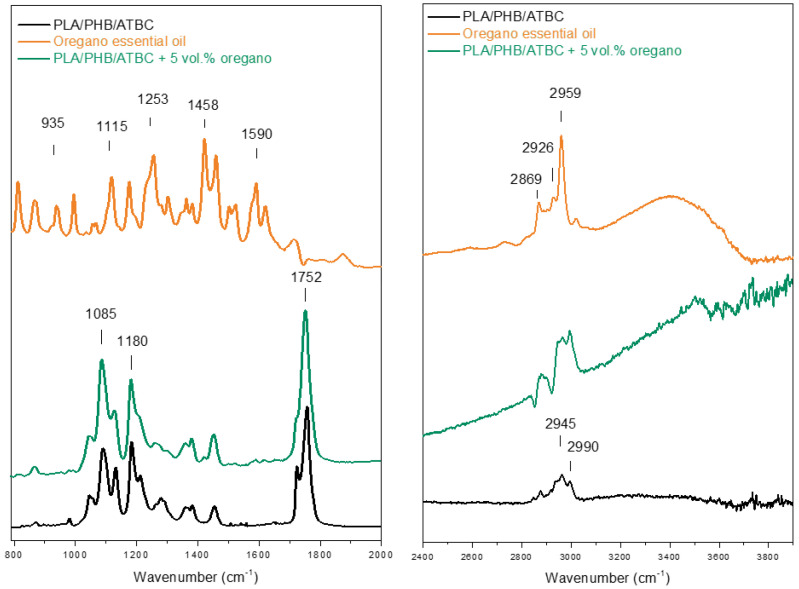
ATR-FTIR spectra of neat PLA/PHB/ATBC, oregano EO, and PLA/PHB/ATBC + 5% oregano EO nanofibrous composite.

**Figure 4:**
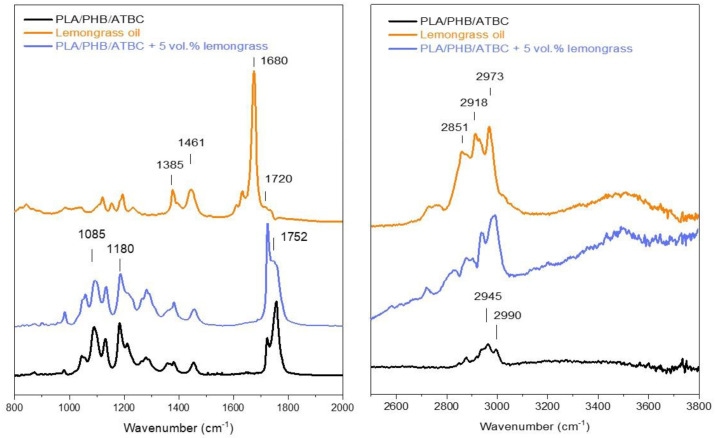
ATR-FTIR spectra of neat PLA/PHB/ATBC, lemongrass EO, and PLA/PHB/ATBC + 5% lemongrass EO nanofibrous composite.

ATR-FTIR spectra of the plain PLA/PHB/ATBC and PLA/PHB/ATBC + 5% oregano EO and PLA/PHB/ATBC + 5% lemongrass EO fibrous are shown in Figure 3 and Figure 4.

The typical FTIR spectra of a plasticized PLA/PHB fibrous web is shown in Figure 3 and Figure 4 by black line. At 1085 cm^−1^, a band associated with the -O-C- asymmetric mode of PLA appears. Meanwhile, at 1180 cm^−1^, a band associated with the -C-O- bond stretching in the -CH-O- group of PLA appears. There is a band at 1278 cm^−1^ assigned to the crystalline -C-O-C stretching of PHB, and at 1360 cm^−1^ the CH deformation and asymmetric bands appear. The typical asymmetric stretching of the carbonyl group (-C=O) in PLA is centered at 1752 cm^−1^, and it is attributed to the amorphous carbonyl vibration. It is possible to observe a small shoulder near 1740 cm^−1^ related to the amorphous state of PHB, showing that the PHB presents mainly crystalline structure with minor amorphous component. The peaks at 2990 and 2945 cm^−1^ clearly are associated with the antisymmetric and symmetric stretching vibrations of CH_3_ of saturated hydrocarbons, respectively [35].

Oregano EO spectra shows peaks at 935 cm^−1^ (CH bending), 1115 cm^−1^ (C-O-C stretching), 1253 cm^−1^ (C-O-C stretching), 1458 cm^−1^ (CH_2_ bending), and 1590 cm^−1^ (N-H bending). Symmetric and asymmetric stretching peaks CH- were revealed at 2869 cm^−1^, 2926 cm^−1^, and 2958 cm^−1^. Spectra exhibited a broad OH stretching peak at around 3380 cm^−1^, attributed to one of the main constituents of oregano oil, carvacrol [36].

Obviously, the presence of oregano oil is confirmed in the fibrous PLA/PHB/ATBC + 5% oregano EO composite as it is seen in Figure 3. The peaks positions of PLA, PHB, and oil did not change after blending with oils, which indicates that there was no chemical interaction between these components.

The FTIR spectra of lemongrass oil having strong characteristic peaks at 3400–3500 cm^−1^ show the presence of OH and peaks at 2918, 2851, and the peak at 1385 cm^−1^ shows the methyl bands; the peak at 1461 cm^−1^ shows the C–H stretching, and a peak at 1680 cm^−1^ shows the vibrations of unsaturated conjugated C=O group present in the oil, confirming the presence of conjugated double bonds in the oil indicates the aldehyde group. The main component of the lemongrass EO is citral [37]. In the spectrum of the oil of lemongrass, some functional groups were observed at 2918 cm^−1^, a predominant asymmetric stretching of -CH_3_ is observed corresponding to an alkyl saturated aliphatic group and at 2851 cm^−1^, and symmetric stretching of -CH_2_ was observed. At the 1461 cm^−1^ peak, the bending of the -CH group is observed. Presence of the lemongrass oil in the fibrous composite can be observed in the wavelength region 2600–3600 cm^−1^. It seems that the peak of C=O group at around 1680 cm^−1^ is shifted in the fibrous PLA/PHB/ATBC + 5% lemongrass EO to 1720 cm^−1^. This may indicate the interaction of oil with polymeric matrix.

### 3.3. TGA

**Figure 5:**
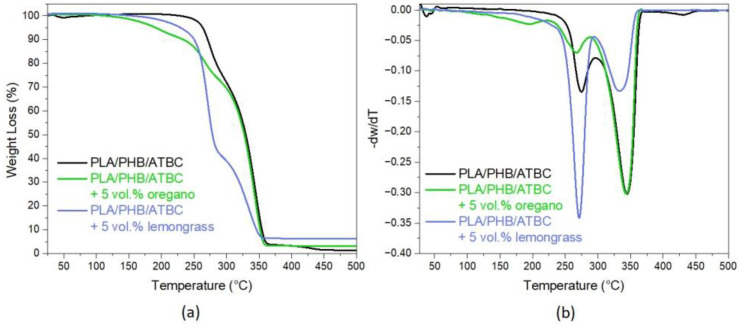
(**a**) TGA thermograms for electrospun neat PLA/PHB/ATBC and composites: PLA/PHB/ATBC + 5% oregano EO and PLA/PHB/ATBC + 5% lemongrass EO. (**b**) Graphs of 1st derivation of TGA.

The thermal stability of all samples was studied by means of thermogravimetric analysis. The results are shown in Figure 5.

The TGA curve of the electrospun plain PLA/PHB/ATBC shows stability up to the temperature of around 230 °C without any weight reduction. PLA/PHB plasticized by ATBC blend was degraded in two steps. The first one, at an initial temperature of 270 °C, the mass reduction was 15%, and it was assigned to the PHB decomposition. The second step, at 340 °C, relates to the PLA thermal degradation. The maximal decomposition was at the temperature of around 450 °C without weight residue. This result agrees with published results when thermal degradation occurs under an inert environment [38].

The addition of the oregano and lemongrass EOs decreases the thermal stability. The PLA/PHB/ATBC + 5% lemongrass EO is stable up to 150 °C without any mass loss. As the temperature increased, lemongrass EO degraded which is in good agreement with the available literature [39]. The degradation temperature in both degradation steps is reduced by about 50 °C compared to the plain PLA/PHB/ATBC. The maximal decomposition was at a temperature of around 420 °C with 5% residue.

On the other hand, the sample PLA/PHB/ATBC + 5% oregano EO was degraded in the three steps. The initial degradation temperature is about 110 °C related to the oregano EO [36], and the second step started at about 230 °C, which is related to the PHB, and the third step started at about 340 °C, which is connected to the PLA. The decomposition temperature was around 370 °C with a minimal residue of roughly 1–2%. By these results, it could be assumed that the blend was not homogenous, which is supported by the observation of emulsion formation.

The first-order derivative, which refers to the temperature where maximum mass decomposition occurred, revealed the two maxima at around 275 °C and 350 °C in the case of plain PLA/PHB/ATBC and PLA/PHB/ATBC + 5% lemongrass EO and three maxima at around 175, 275, and 350 °C in the case of PLA/PHB/ATBC + 5% oregano EO.

### 3.4. Fruit Quality Parameters

#### 3.4.1. Color

Lightness and hue values were measured on time 0, before the application of treatment on strawberry and then through 21 days of storage. The L* and hue values of the fruit skin color were affected by active packaging and storage period (Table 1). Results show a decrease in L* and hue values with storage time for PLA/PHB/ATBC + 5% oregano EO and PLA/PHB/ATBC + 5% lemongrass EO. These changes showed darker fruits through storage. The hue angle is the qualitative attribute of color, which has traditionally been regarded as bluish, yellowish, and reddish. The hue angle indicated that the strawberry treatment by PLA/PHB/ATBC membrane with oregano EO were more reddish, but strawberries after treatment with lemongrass were light reddish. The a* and b* values of treated fruit remained similar with the control during storage, suggesting that coatings had no effect on strawberry color. The a* value was higher on day 7 and then decreased as the storage time progressed. Similar results obtained Ansarifar and Moradinezhad [21], who stated that this is related to the reduction of anthocyanin content in strawberry fruit during storage. Chroma is the characteristic that allows the assessment of intensity/purity of any given hue, and it is considered the quantitative attribute of color. Chroma values did not show significant changes among treatments and control. Such results indicate that active membrane packaging with oregano EO have a greater effect on hue retention, thus slowing the ripening process.

#### 3.4.2. Firmness

Fruit firmness is an important factor for determination of the postharvest quality of strawberries. Firmness, as measured by puncture with a 7 mm diameter probe, decreased in all fruits through storage period and shelf-life (Table 1). However, the firmness of all strawberries slowly reduced after 14 days of storage. The samples packaged with PLA/PHB membrane with oregano showed the highest firmness values through the experiment, with suitable firmness values for consumption, while the samples packaged by PLA/PHB/ATBC + 5% lemongrass EO and the control fruits were too soft and close to being overripe at the end of the experiment. The results suggest that oregano EO released from the nanofiber maintained the cell wall integrity of the fruit and reduced moisture loss, consistent with similar previous studies [40]. According to Min et al. [10], softening and loss of firmness was significantly reduced in strawberries when packaged in PLA with EOs.

#### 3.4.3. Soluble Solids Content

The SSC, measured as % ºBrix, was 7.15% in the fruit juice at harvest and decreased during storage in all treatments and control (Table 1). Specifically, the highest decrease was observed in control samples. After 3 weeks of cold storage, treated strawberries had similar contents of SSC as the control samples. Overall reduction might be due to sugar metabolism during the respiratory process [41].

#### 3.4.4. Weight Loss

The loss of weight in fruits is associated with respiration rate and evaporation of moisture through the skin. The rapid loss of water from the skin is one of the major factors that contributes to the perishability of strawberry fruits [42]. Weight loss, expressed as percentage of initial fruit weight, was noticed in all fruits upon storage and shelf-life (Table 1). Control fruit had the higher weight loss during storage. Samples treated by PLA/PHB/ATBC membrane with EOs had significantly lower weight loss than control during all times tested. The lowest weight loss (3.98%) was obtained in active packaging with PLA/PHB/ATBC + 5% lemongrass EO. Comparing treatments with control, lemongrass and oregano EOs released from the PLA/PHB/ATBC films slowed the ripening process and significantly reduced weight loss of fruit. Similar results were obtained in research by Amal et al. [43], who detected that strawberry packaged in polymer foil with thyme EO significantly reduced fruit weight loss. However, there was a significant increase in weight loss through storage in treated and non-treated strawberries. The highest fruit weight loss was detected after 3 weeks of storage. According to Dhital et al. [44], in a study on strawberry, the highest weight loss was observed on the 14th day. We can assume that the fruit weight loss occurred based on respiratory metabolic process and water evaporation during storage. However, the weight loss of packed fruits with PLA/PHB/ATBC film with EO was significantly reduced. Generally, the lower weight losses lead to longer storage life and freshness.

### 3.5. Total Phenolic Content

Natural plant antioxidants, such as phenolic compounds, may produce beneficial effects by scavenging free radicals [19,45]. The total phenolic content of fresh strawberry, in our case in free form, was 86.28 mg GAE g^−1^ before storage (Table 1). Through time, mainly in the first 7 days of storage, an increasing trend on total phenolic content of strawberries was noticed, except for control. The highest amount was detected in strawberries packaged by PLA/PHB/ATBC + 5% oregano EO. However, after 2 weeks of storage, it was observed that the amount of total phenolic content had no significant differences among treated and non-treated fruit. Significant increase in the total phenolic content in the first days after treatment with EO was described by Nunes et al. [46] and Dhital et al. [44]. The increase in the phenolic content of strawberries during storage can be attributed to the accumulation of anthocyanins and the development of its dark red color [44].

### 3.6. Antioxidant Activity

Several studies have indicated that phenolic contents are strongly related to antioxidant activity. According to Gülçin et al. [47], a high level of phenolic compounds indicates the elevated antioxidant capacity of EO. According to Wang et al. [48], antioxidants can reduce the physiological deterioration of fruits during storage, which reinforces the behavior found in our study. The antioxidant capacity of the strawberry was determined by the DPPH method, and the results are shown in Table 1. There was not a significant change in antioxidant activity measured by DPPH method among treatments or through storage time.

### 3.7. Total Anthocyanin Content

According to Dhital et al. [44], anthocyanins are responsible for the characteristic red color of ripe strawberries. During storage, the level of anthocyanins increased in all fruit (Table 1). With the exception of control after 7 days of storage, with lower values, there were no significant differences among treatments in anthocyanin content.

Although not statistically significant, strawberries treated by PLA/PHB/ATBC + 5% lemongrass EO (33.76 mg 100 g FW) showed the highest values of anthocyanins at the end of the experiment. According to Cordenunsi et al. [49] and Colussi et al. [19], after strawberry harvest and during storage, the anthocyanin biosynthesis process remains active, even at low temperatures. 

### 3.8. Aerobic Mesophilic Bacteria, Psychrotrophic Bacteria, and Yeasts and Molds

For antimicrobial active packaging materials, the antimicrobial activity is a very important property. Fruits in packages with PLA/PHB/ATBC with EOs showed lower microbial development that control, with the effect more evident on yeasts and molds (Table 2). In our previous study, we reported that some EOs have antimicrobial action [13,14]. Adding EOs to active packaging improves its antimicrobial action [3]. This assertion was confirmed in our study, and we can evaluate that strawberries treated by PLA/PHB/ATBC membrane with EOs achieved lower contamination by yeasts and molds during storage. However, the best antifungal activity was determined with PLA/PHB/ATBC + 5% lemongrass EO. The PLA/PHB/ATBC with oregano or lemongrass EO with their antifungal activity reduce reproduction of fungi. 

Nevertheless, according to Bierhals et al. [50], the limit of acceptance for the consumption of fruit products is 6 Log CFU/g. In our study, the counts of yeasts, molds, and mesophilic aerobic microorganisms did not reach that limit during storage. In the case of psychrophilic aerobic bacteria, no microorganism growth was found. According to our results, these PLA/PHB/ATBC membranes with EOs showed good antimicrobial activities.

We tested application of PLA/PHB/ATBC membranewith EOs not just by evaporation but also by direct contact with strawberries. Direct contact caused faster rotting in all tested strawberries treated by pure PLA/PHB/ATBC membrane and the fastest after being treated by PLA/PHB/ATBC with EOs compared with control (data not shown). Compounds of polymers with EOs at higher concentrations can have harmful effects after the contact with the fruit tissue and damage the cellular tissues irreparably. The fruit does not have enough time to increase the antioxidant activity in its tissues with respect to the oxygen uptake capacity and free radical scavenging ability [19,48,51].

### 3.9. Sensory Evaluation

EO-based packaging has been applied to food products to extend their shelf-life by keeping or improving their appearance, flavor, aroma, as well as nutritional quality [52,53]. However, they can change their sensory properties [20]. As the final consumer decides whether treated fruits are acceptable, sensory evaluation is required [54]. In our work, after 18 days of storage, the appearance, which is the first condition for consumers’ decision to buy, was with a good sensory appreciation (≥6.0 in a scale of 1-dislike to 7-like definitely) for all fruits—specifically 6.3 for control and fruits treated by PLA/PHB with oregano and 6.0 for fruits treated by PLA/PHB/ATBC + 5% lemongrass EO. (Table 3). For all others evaluating parameters (texture, sweetness, and acidity, aroma), controls achieved lower evaluation than treated fruit. According to taste panels, fruits treated by PLA/PHB/ATBC + 5% lemongrass EO achieved higher scores for aroma and texture in comparison to other treated and non-treated fruits. On the other side, for sweetness and acidity, the best results were obtained in fruits treated with PLA/PHB/ATBC + 5% oregano EO. Overall liking was better in fruit packed in PLA/PHB/ATBC + 5% lemongrass EO, and the lowest appreciated were non-treated strawberries (Table 3). A very important observation is that the aroma of strawberries was not negatively affected by the treatment with EOs. Overall, that the sensory properties of fruit, especially strawberries, improved through active packaging including EOs was also reported by previous studies [2,54,55,56].

## 4. Conclusions

In this study, PLA/PHB/ATBC + EO active packaging membranes were developed by electrospinning. FTIR proved the presence of EOs in electrospun membranes due to the presence of EOs characteristic peaks. SEM observation displayed high porosity of developed membranes, which allows the permeability of gasses such as oxygen and carbon dioxide. Thermogravimetric analysis revealed that thermal stability of membranes was affected by EOs negatively with the decomposition temperature decreasing, but stability it is still adequate to fulfill purposes related to food protection during their use, storage, or transport. The developed membranes were used for active packaging of strawberries. The fruit quality parameters (color, firmness, SSC, weight loss, total phenolic content, antioxidant activity, total anthocyanin content) indicated that active membrane packaging with EOs slowed down the ripening process, maintained the cell wall integrity of the fruit, reduced moisture loss, and caused the lower weight losses to lead to longer storage life and freshness. The results of this study also indicated that the strawberries stored in packaging with PLA/PHB/ATBC + EOs with antifungal activity had reduced fungal growth and showed good antimicrobial activities. At the end of the storage period, strawberries in active packaging showed greater acceptance and quality characteristics than the fruits packaged only with the PLA/PHB/ATBC membrane. According to the sensory properties of the fruit, overall liking was better for the fruits packed in PLA/PHB/ATBC + 5% lemongrass EOs. The utilization of active packaging by PLA/PHB/ATBC + 5% lemongrass EOs is an appropriate tool to preserve the quality and reduce post-harvest deterioration of strawberries. Active packaging by PLA/PHB/ATBC + 5% oregano EOs could be more fitted for packaging and shelf-life extension of other fruits or vegetables. Therefore, it is possible to consider our attempt as successful, and different combinations of biodegradable polymers and antimicrobial natural compounds can be tried in the future in order to produce dedicated active packaging for other food products.

## Figures and Tables

**Table 1:** Changes in quality parameters of strawberry for 3 weeks of storage for control and treatment by PLA/PHB/ATBC + 5% oregano EO and PLA/PHB/ATBC + 5% lemongrass EO.

Quality Parameters	Treatments	Storage Duration (Days)			
		0	7	14	21
Lightness (L*)	Control	42.06 ± 1.99 ^aA^	40.01 ± 1.65 ^bB^	40.58 ± 1.44 ^aB^	39.85 ± 1.21 ^aB^
	PLA/PHB/ATBC + 5% oregano EO	42.06 ± 1.99 ^aA^	42.26 ± 0.57 ^aA^	39.86 ± 1.05 ^abB^	39.14 ± 0.87 ^aB^
	PLA/PHB/ATBC + 5% lemongrass EO	42.06 ± 1.99 ^aA^	39.12 ± 0.70 ^bB^	39.04 ± 0.96 ^bB^	37.54 ± 0.37 ^bB^
a*	Control	32.49 ± 1.45 ^aB^	34.46 ± 0.72 ^bA^	34.25 ± 0.95 ^aA^	33.98 ± 1.08 ^aAB^
	PLA/PHB/ATBC + 5% oregano EO	32.49 ± 1.45 ^aB^	35.51 ± 0.52 ^abA^	33.78 ± 0.34 ^aAB^	33.24 ± 0.11 ^aB^
	PLA/PHB/ATBC + 5% lemongrass EO	32.49 ± 1.45 ^aC^	35.85 ± 0.22 ^aA^	34.41 ± 0.57 ^aB^	33.64 ± 1.08 ^aBC^
b*	Control	24.71 ± 2.14 ^aA^	25.16 ± 1.56 ^bA^	25.04 ± 1.86 ^aA^	24.58 ± 1.42 ^aA^
	PLA/PHB/ATBC + 5% oregano EO	24.71 ± 2.14 ^aB^	28.06 ± 0.57 ^aA^	24.20 ± 0.89 ^aB^	24.40 ± 1.70 ^aB^
	PLA/PHB/ATBC + 5% lemongrass EO	24.71 ± 2.14 ^aA^	24.83 ± 1.08 ^bA^	23.04 ± 1.26 ^aA^	25.10 ± 5.50 ^aA^
Hue angle (h)	Control	36.81 ± 1.46 ^aA^	36.02 ±1.54 ^bA^	35.85 ± 1.65 ^abA^	35.72 ± 0.70 ^aA^
	PLA/PHB/ATBC + 5% oregano EO	36.81 ± 1.46 ^aA^	38.00 ± 0.75 ^aA^	35.27 ± 0.79 ^bA^	37.07 ± 3.62 ^aA^
	PLA/PHB/ATBC + 5% lemongrass EO	36.81 ± 1.46 ^aA^	34.51 ± 1.18 ^bAB^	33.64 ± 1.12 ^aC^	34.64 ± 2.48 ^aAB^
Chroma	Control	41.01 ± 2.47 ^aA^	42.73 ± 1.31 ^bA^	42.37 ± 1.83 ^aA^	41.98 ± 1.71 ^aA^
	PLA/PHB/ATBC + 5% oregano EO	41.01 ± 2.47 ^aB^	45.38 ± 0.51 ^aA^	41.64 ± 0.68 ^aB^	41.04 ± 0.74 ^aB^
	PLA/PHB/ATBC + 5% lemongrass EO	41.01 ± 2.47 ^aA^	43.68 ± 0.68 ^abA^	41.48 ± 1.09 ^aA^	42.77 ± 4.92 ^aA^
Firmness (N)	Control	6.54 ± 0.35 ^aAB^	7.06 ± 0.23 ^abA^	5.89 ± 0.23 ^aB^	4.45 ± 0.62 ^aC^
	PLA/PHB/ATBC + 5% oregano EO	6.54 ± 0.35 ^aB^	7.97 ± 1.71 ^aA^	6.20 ± 1.09 ^aB^	4.64 ± 0.81 ^aC^
	PLA/PHB/ATBC + 5% lemongrass EO	6.54 ± 0.35 ^aA^	6.42 ± 0.84 ^bA^	4.77 ± 0.50 ^bB^	3.32 ± 1.29 ^bC^
SSC (%)	Control	7.15 ± 0.45 ^aA^	6.20 ± 0.55 ^aB^	6.23 ± 0.28 ^aB^	5.70 ± 0.37 ^aB^
	PLA/PHB/ATBC + 5% oregano EO	7.15 ± 0.45 ^aA^	6.75 ± 0.17 ^aA^	6.28 ± 1.28 ^aA^	6.08 ± 0.30 ^aA^
	PLA/PHB/ATBC + 5% lemongrass EO	7.15 ± 0.45 ^aA^	6.35 ± 0.59 ^aB^	6.10 ± 0.39 ^aB^	6.13 ± 0.39 ^aB^
Weight loss (%)	Control		2.19 ± 0.10 ^aC^	3.80 ± 0.22 ^aB^	5.42 ± 0.24 ^aA^
	PLA/PHB/ATBC + 5% oregano EO		1.50 ± 0.16 ^bC^	2.73 ± 0.34 ^bB^	4.27 ± 0.42 ^bA^
	PLA/PHB/ATBC + 5% lemongrass EO		1.44 ± 0.06 ^bC^	2.54 ± 0.14 ^bB^	3.98 ± 0.24 ^bA^
Total phenolic content (mg GAE.g^−1^)	Control	86.28 ± 8.58 ^aC^	79.05 ± 11.84 ^bC^	233.51 ± 26.15 ^aB^	266.27 ± 17.47 ^aA^
	PLA/PHB/ATBC + 5% oregano EO	86.28 ± 8.58 ^aB^	246.88 ± 91.56 ^aA^	220.32 ± 33.87 ^aA^	194.24 ± 102.46 ^aAB^
	PLA/PHB/ATBC + 5% lemongrass EO	86.28 ± 8.58 ^aB^	191.73 ± 75.47 ^aA^	201.28 ± 43.65 ^aA^	268.92 ± 69.43 ^aA^
Antioxidant activity (µM Trolox.g^−1^ FW)	Control	172.86 ± 10.97 ^aA^	166.97 ± 14.76 ^aA^	167.34 ± 11.63 ^aA^	154.80 ± 10.52 ^aA^
	PLA/PHB/ATBC + 5% oregano EO	172.86 ± 10.97 ^aA^	176.8 ± 7.86 ^aA^	175.18 ± 12.00 ^aA^	161.98 ± 8.45 ^aA^
	PLA/PHB/ATBC + 5% lemongrass EO	172.86 ± 10.97 ^aA^	164.26 ± 12.17 ^aA^	158.5 ± 16.98 ^aA^	150.54 ± 11.23 ^aA^
Total anthocyanin content (mg 100 g FW)	Control	16.57 ± 3.04 ^aB^	9.53 ± 2.30 ^bC^	29.15 ± 1.47 ^aA^	29.49 ± 2.76 ^aA^
	PLA/PHB/ATBC + 5% oregano EO	16.57 ± 3.04 ^aB^	17.31 ± 4.61 ^aB^	27.59 ± 3.92 ^aA^	21.55 ± 8.83 ^aA^
	PLA/PHB/ATBC + 5% lemongrass EO	16.57 ± 3.04 ^aB^	16.69 ± 5.40 ^aB^	30.39 ± 7.37 ^aA^	33.76 ± 8.91 ^aA^

Data shown are the mean ± standard error of four replicates with 10 fruits each. The values followed by the same lower-case letter, in the same column and parameter and by the same uppercase letter in the same row are not significantly different (Duncan’s new multiple range test at *p* < 0.05).

**Table 2:** Effect of active packaging of strawberry by PLA/PHB/ATBC + 5% oregano EO and PLA/PHB/ATBC + 5% lemongrass EO on yeast and molds and aerobic mesophilic bacteria during storage.

Microorganisms (Log CFU/g)	Treatments	Storage Duration (days)			
		0	7	14	21
Yeast and molds	Control	1.09 ± 0.11 ^aB^	2.36 ± 0.25 ^aA^	1.27 ± 0.85 ^aAB^	1.04 ± 1.21 ^aB^
	PLA/PHB/ATBC + 5% oregano EO	1.09 ± 0.11 ^aA^	1.35 ± 0.91 ^abA^	1.27 ± 0.85 ^aA^	0.42 ± 0.85 ^aA^
	PLA/PHB/ATBC + 5% lemongrass EO	1.09 ± 0.11 ^aA^	0.92 ± 1.08 ^bA^	0.00 ± 0.00 ^bB^	0.00 ± 0.00 ^aB^
Aerobic mesophilic bacteria	Control	0.84 ± 0.57 ^aA^	0.67 ± 0.47 ^aA^	0.89 ± 0.24 ^abA^	1.28 ± 0.21 ^abA^
	PLA/PHB/ATBC + 5% oregano EO	0.84 ± 0.57 ^aA^	0.35 ± 0.40 ^aA^	0.75 ± 0.50 ^bA^	0.94 ± 0.63 ^bA^
	PLA/PHB/ATBC + 5% lemongrass EO	0.84 ± 0.57 ^aA^	1.32 ± 0.98 ^aA^	1.34 ± 0.23 ^aA^	1.65 ± 0.18 ^aA^

Data shown are the mean ± standard error of four independent samples consisting of ten replicates each. The values followed by the same lowercase letter in the same column and parameter and by the same uppercase letter in the same row are not significantly different (Duncan’s new multiple range test at *p* < 0.05).

**Table 3:** Sensory evaluation of strawberries in active packaging using PLA/PHB/ATBC + 5% oregano EO and PLA/PHB/ATBC + 5% lemongrass EO after 18 days of storage.

Taste Panels	Treatments	Evaluation
Fruit appearance	Control	6.3 ± 0.73
	PLA/PHB/ATBC + 5% oregano EO	6.3 ± 0.66
	PLA/PHB/ATBC + 5% lemongrass EO	6.0 ± 0.80
Aroma	Control	5.15 ± 0.99
	PLA/PHB/ATBC + 5% oregano EO	5.25 ± 1.16
	PLA/PHB/ATBC + 5% lemongrass EO	5.90 ± 0.91
Texture	Control	5.70 ± 0.92
	PLA/PHB/ATBC + 5% oregano EO	5.75 ± 1.07
	PLA/PHB/ATBC + 5% lemongrass EO	5.85 ± 1.14
Sweetness	Control	4.7 ± 1.26
	PLA/PHB/ATBC + 5% oregano EO	5.25 ± 1.48
	PLA/PHB/ATBC + 5% lemongrass EO	5.20 ± 1.40
Acidity	Control	5.05 ± 1.00
	PLA/PHB/ATBC + 5% oregano EO	5.45 ± 1.40
	PLA/PHB/ATBC + 5% lemongrass EO	5.35 ± 1.31
Flavor in general	Control	5.15 ± 0.88
	PLA/PHB/ATBC + 5% oregano EO	5.35 ± 1.46
	PLA/PHB/ATBC + 5% lemongrass EO	5.80 ± 1.15

Data shown are the mean ± standard error of four independent samples consisting of ten replicates each.

## Data Availability

Data are contained within the article.

## Supplementary Materials

The following supporting information can be downloaded at: https://www.mdpi.com/article/10.3390/antiox12030755/s1.
