# Supplementary material for: Biodegradable Active Packaging Enriched with Essential Oils for Enhancing the Shelf Life of Strawberries
Source: Antioxidants (Basel). 2023 Mar 20;12(3):755. doi: 10.3390/antiox12030755 (PMC10044849; doi:10.3390/antiox12030755)
Supplement: Supplementary file 1 [file antioxidants-12-00755-s001.zip › Lemongrass-2111313.pdf]

Aromatic Plant Research Center

We provide uncompromising
quality control for your products.

Customer : doTERRA International
Lot Number : 2111313
Date Filled : 04/23/2021

Column : ZB5 (60 m length × 0.25 mm inner diameter × 0.25 μm film thickness)
Instrument : Shimadzu GCMS-QP2010 Ultra
Carrier gas : Helium 80 psi
Temperature ramp : 2 degrees celsius per minute up to 260-degrees celsius
Split ratio : 30:1
Sample preparation : 5%w/v solution with Dichloromethane.

Comments:

The analysis of this Lemongrass lot revealed no contaminants or adulteration.
The sample meets the expected chemical profile for authentic essential oils of Cymbopogon flexuosus.

Analysied by : Ambika Poudel
Reviewed by : Dr. Prabodh Satyal

CERTIFIED BY AROMATIC PLANT RESEARCH CENTER

© Copyright 2021 Aromatic Plant Research Center. All rights reserved. Any publishing, copying, use, dissemination, or distribution of this report, including online, without the express written permission of Aromatic Plant Research Center is strictly prohibited.

Aromatic Plant Research Center
230 N 1200 E STE 100
Lehi, UT 84043
www.aromaticplant.org

APRC
Aromatic Plant Research Center

Lemongrass Essential Oil

Customer : doTERRA International

Lot Number : 2111313

Date Filled : 04/23/2021

Chromatogram

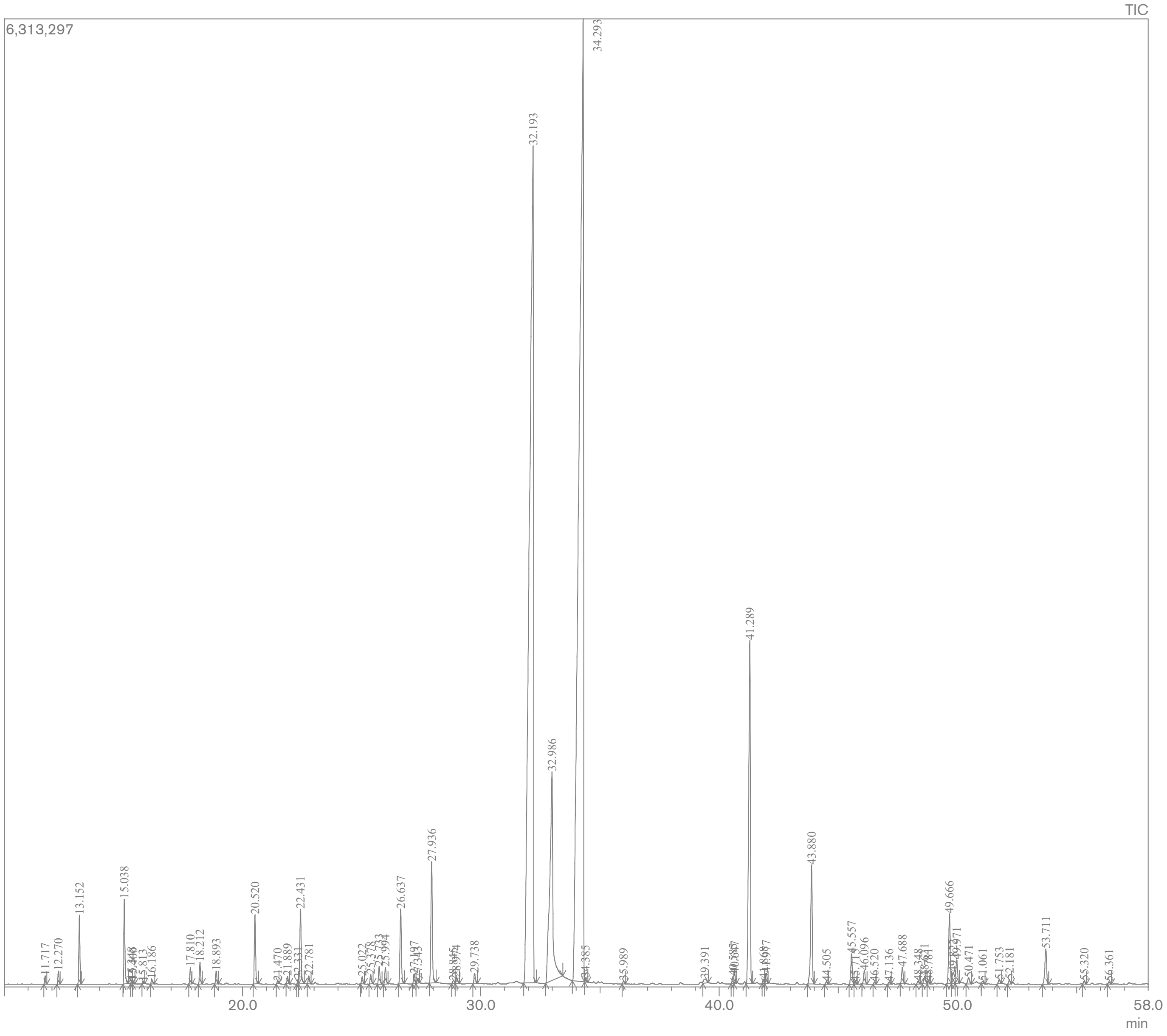

© Copyright 2021 Aromatic Plant Research Center. All rights reserved. Any publishing, copying, use, dissemination, or distribution of this report, including online, without the express written permission of Aromatic Plant Research Center is strictly prohibited.

Aromatic Plant Research Center
230 N 1200 E STE 100
Lehi, UT 84043
www.aromaticplant.org

APRC® Aromatic Plant Research Center

Peak Report

Peak#	R.Time	Name	Area%
1	11.717	Tricyclene	0.08
2	12.270	alpha-Pinene	0.12
3	13.152	Camphene	0.69
4	15.038	6-Methyl-5-hepten-2-one	0.90
5	15.348	Myrcene	0.04
6	15.400	Dehydro-1,8-Cineole	0.03
7	15.813	trans-Dehydroxy linalool oxide	0.02
8	16.186	n-Octanal	0.06
9	17.810	Limonene	0.19
10	18.212	cis-beta-Ocimene	0.25
11	18.893	trans-beta-Ocimene	0.15
12	20.520	2-Nonanone	0.82
13	21.470	Terpinolene	0.03
14	21.889	Rosefuran	0.09
15	22.331	Perillene	0.05
16	22.431	Linalool	0.98
17	22.781	alpha-Pinene oxide	0.10
18	25.022	Epiphotocitral A	0.09
19	25.378	exo-Isocitral	0.13
20	25.733	trans-Chrysanthanal	0.23
21	25.994	Citronellal	0.22
22	26.637	cis-Isocitral	1.00
23	27.197	Rosefuran epoxide	0.13
24	27.343	Borneol	0.06
25	27.936	trans-Isocitral	1.68
26	28.845	Oxy citral	0.06
27	28.974	alpha-Terpineol	0.10
28	29.738	n-Decanal	0.15
29	32.193	Neral	32.05
30	32.986	Geraniol	6.42
31	34.293	Geranial	41.27
32	34.385	4-Undecanone	0.05
33	35.989	Geranyl formate	0.04
34	39.391	Neric acid	0.11
35	40.585	Cyclosativene	0.10
36	40.647	alpha-Ylangene	0.13
37	41.289	Geranyl acetate	5.51
38	41.868	beta-Cubebene	0.06
39	41.977	beta-Elemene	0.16
40	43.880	beta-Caryophyllene	1.83
41	44.505	beta-Copaene	0.04
42	45.557	trans-Isoeugenol	0.45
43	45.715	trans-Muurola-3,5-diene	0.04
44	46.096	alpha-Humulene	0.20
45	46.520	cis-Muurola-4(14),5-diene	0.03
46	47.136	trans-Cadina-1(6),4-diene	0.03
47	47.688	Germacrene D	0.24
48	48.348	trans-Muurola-4(14),5-diene	0.04
49	48.621	epi-Cubebol	0.14
50	48.781	alpha-Muurolene	0.04
51	49.666	gamma-Cadinene	1.05
52	49.822	Cubebol	0.23
53	49.971	delta-Cadinene	0.39
54	50.471	trans-gamma-Bisabolene	0.11
55	51.061	alpha-Cadinene	0.03
56	51.753	alpha-Elemol	0.06
57	52.181	Geranyl butanoate	0.06
58	53.711	Caryophyllene oxide	0.60
59	55.320	Humulene epoxide II	0.05
60	56.361	1-epi-Cubenol	0.03
			100.00

CERTIFIED BY AROMATIC PLANT RESEARCH CENTER

© Copyright 2021 Aromatic Plant Research Center. All rights reserved. Any publishing, copying, use, dissemination, or distribution of this report, including online, without the express written permission of Aromatic Plant Research Center is strictly prohibited.
