# Supplementary material for: Biodegradable Active Packaging Enriched with Essential Oils for Enhancing the Shelf Life of Strawberries
Source: Antioxidants (Basel). 2023 Mar 20;12(3):755. doi: 10.3390/antiox12030755 (PMC10044849; doi:10.3390/antiox12030755)
Supplement: Supplementary file 1 [file antioxidants-12-00755-s001.zip › Oregano-211265.pdf]

Aromatic Plant Research Center

We provide uncompromising
quality control for your products.

Customer : doTERRA International
Lot Number : 211265
Date Filled : 05/06/2021

Column : ZB5 (60 m length × 0.25 mm inner diameter × 0.25 μm film thickness)
Instrument : Shimadzu GCMS-QP2010 Ultra
Carrier gas : Helium 80 psi
Temperature ramp : 2 degrees celsius per minute up to 260-degrees celsius
Split ratio : 30:1
Sample preparation : 5%w/v solution with Dichloromethane.

Comments:
The analysis of this Oregano lot revealed no contaminants or adulteration.
The sample meets the expected chemical profile for authentic essential oils of Origanum vulgare.

Analysied by : Ambika Poudel
Reviewed by : Dr. Prabodh Satyal

CERTIFIED BY
AROMATIC PLANT RESEARCH CENTER

© Copyright 2021 Aromatic Plant Research Center. All rights reserved. Any publishing, copying, use, dissemination, or distribution of this report, including online, without the express written permission of Aromatic Plant Research Center is strictly prohibited.

Aromatic Plant Research Center
230 N 1200 E STE 100
Lehi, UT 84043
www.aromaticplant.org

APRC®
Aromatic Plant Research Center

Oregano Essential Oil

Customer : doTERRA International

Lot Number : 211265

Date Filled : 05/06/2021

Chromatogram

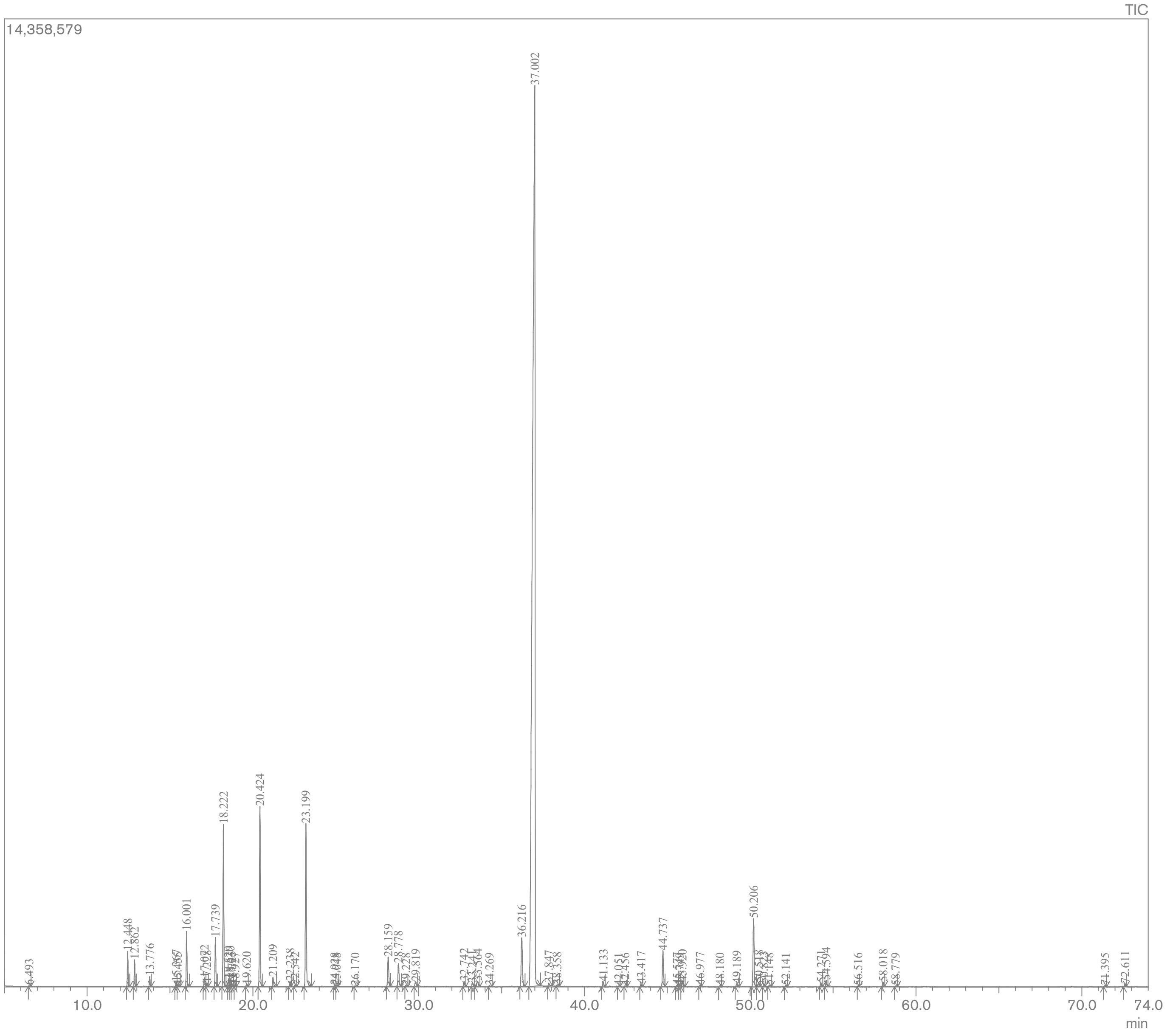

CERTIFIED BY AROMATIC PLANT RESEARCH CENTER

© Copyright 2021 Aromatic Plant Research Center. All rights reserved. Any publishing, copying, use, dissemination, or distribution of this report, including online, without the express written permission of Aromatic Plant Research Center is strictly prohibited.

Aromatic Plant Research Center
230 N 1200 E STE 100
Lehi, UT 84043
www.aromaticplant.org

APRC®
Aromatic Plant Research Center

Peak Report

Peak#	R.Time	Name	Area%
1	6.493	Methyl-alpha-methyl butyrate	0.02
2	12.448	alpha-Thujene	0.78
3	12.862	alpha-Pinene	0.60
4	13.776	Camphene	0.24
5	15.367	beta-Pinene	0.10
6	15.455	1-Octen-3-ol	0.07
7	16.001	Myrcene	1.35
8	17.072	alpha-Phellandrene	0.23
9	17.228	delta-3-Carene	0.09
10	17.739	alpha-Terpinene	1.27
11	18.222	para-Cymene	4.23
12	18.529	Limonene	0.20
13	18.630	beta-Phellandrene	0.21
14	18.725	1,8-cineole	0.06
15	18.927	cis-beta-Ocimene	0.04
16	19.620	trans-beta-Ocimene	0.07
17	20.424	gamma-Terpinene	4.89
18	21.209	cis-Sabinene hydrate	0.31
19	22.238	Terpinolene	0.16
20	22.542	para-Cymenene	0.05
21	23.199	Linalool	4.91
22	24.928	cis-para-Menth-2-en-1-ol	0.04
23	25.048	alpha-Campholenal	0.04
24	26.170	trans-para-Menth-2-en-1-ol	0.03
25	28.159	Borneol	0.96
26	28.778	Terpinen-4-ol	0.78
27	29.228	para-Cymen-8-ol	0.04
28	29.819	alpha-Terpineol	0.18
29	32.742	Methyl carvacrol ether	0.16
30	33.241	Carvone	0.13
31	33.564	Linalyl acetate	0.24
32	34.269	Carvenone	0.04
33	36.216	Thymol	1.84
34	37.002	Carvacrol	70.30
35	37.847	Methyl non-8-enoate	0.13
36	38.358	tert-Butyl-para-benzoquinone	0.05
37	41.133	Carvacryl acetate	0.15
38	42.051	Geranyl acetate	0.03
39	42.456	beta-Bourbonene	0.03
40	43.417	Methyleugenol	0.09
41	44.737	beta-Caryophyllene	1.18
42	45.577	trans-alpha-Bergamotene	0.06
43	45.749	TertButylcatechol	0.03
44	45.920	Aromadendrene	0.16
45	46.977	alpha-Humulene	0.06
46	48.180	trans-Cadina-1(6),4-diene	0.04
47	49.189	Viridiflorene	0.11
48	50.206	beta-Bisabolene	2.21
49	50.518	gamma-Cadinene	0.15
50	50.823	delta-Cadinene	0.10
51	51.148	beta-Sesquiphellandrene	0.04
52	52.141	trans-alpha-Bisabolene	0.04
53	54.271	Spathulenol	0.13
54	54.594	Caryophyllene oxide	0.21
55	56.516	1,10-Di-epi-cubenol	0.02
56	58.018	alpha-Muurolol	0.16
57	58.779	alpha-Cadinol	0.03
58	71.395	Oregano Sesquiterpenoid 1	0.05
59	72.611	Oregano Sesquiterpenoid 2	0.10
			100.00

CERTIFIED BY AROMATIC PLANT RESEARCH CENTER

© Copyright 2021 Aromatic Plant Research Center. All rights reserved. Any publishing, copying, use, dissemination, or distribution of this report, including online, without the express written permission of Aromatic Plant Research Center is strictly prohibited.
